# Accuracy of a continuous non-invasive hemoglobin monitor in the ICU

**DOI:** 10.1186/cc9849

**Published:** 2011-03-11

**Authors:** D Frasca, C Dahyot-Fizelier, C Karen, Q Levrat, N Soumagne-Vialle, M Boisson, B Debaene, O Mimoz

**Affiliations:** 1 CHU Poitiers, France

## Introduction

The non-invasive pulse CO-oximeter provides an immediate and continuous estimation of hemoglobin concentration non-invasively, and so has the potential to improve ICU patient care. We determined whether non-invasive hemoglobin measurement by pulse CO-oximetry could provide clinically acceptable absolute and trend accuracy in critically ill patients, compared with other invasive methods of hemoglobin assessment available at bedside and the gold standard, the laboratory analyzer.

## Methods

A prospective study was conducted in the surgical ICU of a university teaching hospital. Blood samples from subjects continuously monitored with pulse CO-oximetry (SpHb) were analyzed for hemoglobin concentration determination by a point-of-care device (HemoCue301, HbHC), satellite laboratory CO-oximetry (Siemens RapidPoint 450, HbABG) and a laboratory hematology analyzer (Sysmex XT-2000i, tHB), which was considered the reference device. Hemoglobin values reported from the invasive methods were compared with the values reported by the Masimo Radical-7 Pulse CO-Oximeter at the time of the blood draw.

## Results

Sixty-two patients requiring 471 blood samples were included. Compared with the reference method, the bias and limits of agreement were 0.0 ± 1.0 g/dl for SpHb, 0.3 ± 1.3 g/dl for HbHC and 0.9 ± 0.6 g/dl for HbABG compared with the reference device. Accuracy assessed with ARMS was 0.8 g/dl for SpHb and 1.1 g/dl for HbABG and HbHC. Pulse CO-oximetry showed similar trend accuracy as CO-oximetry, whereas the point-of-care device did not follow the trend of the laboratory device as well as the other analyzers. See Table 1.

**Table 1:** Accuracy summary of test devices compared with laboratory hematology analyzer

	SpHb vs. tHb	HbHC vs. tHb	HbABG vs. tHb
Bias (g/dl)	0.0	-0.3	-0.9
Agreement(g/dl)	-1.0 to 0.9	-1.6 to 1.0	-1.6 to -0.3
ARMS (g/dl)	0.8	1.1	1.1

## Conclusions

When compared with laboratory reference values, hemoglobin measurement with pulse CO-oximetry has absolute and trending accuracy similar to widely used, invasive methods such as CO-oximetry and a point-of-care device. Hemoglobin measurement with pulse CO-oximetry has the additional advantages of providing continuous measurements, non-invasively, which may facilitate hemoglobin monitoring in the ICU.

